# Millimeter-Wave MIMO radar for contactless neonatal heart-rate assessment: performance under common resuscitation-related maneuvers and feasibility in clinical workflow

**DOI:** 10.3389/fped.2026.1841964

**Published:** 2026-06-12

**Authors:** Xuerui Liang, Qingqing Wang, Zheng Yan, Kunhong Lin, Tingyue Hu, Xiangwei Dang, Nanyi Jiang, Shiguang Yang, Yanlei Li, Tongyan Han

**Affiliations:** 1 Department of Pediatrics, Peking University Third Hospital, Beijing, China; 2 School of Information Science and Technology, North China University of Technology, Beijing, China; 3 National Key Laboratory of Microwave Imaging Technology, Aerospace Information Research Institute, Chinese Academy of Sciences, Beijing, China; 4 School of Electronic, Electrical and Communication Engineering, University of Chinese Academy of Sciences, Beijing, China

**Keywords:** heart rate, neonatal monitoring, neonatal resuscitation, non-contact monitoring, radar

## Abstract

**Background:**

Rapid and reliable heart-rate (HR) assessment is critical during neonatal resuscitation. Contactless monitoring may serve as a valuable adjunct to conventional methods, but the clinical performance of millimeter-wave radar under routine resuscitation-related maneuvers remains unclear. We evaluated the performance and workflow feasibility of a millimeter-wave multiple-input multiple-output (MIMO) radar system for contactless neonatal HR assessment.

**Methods:**

In this single-center prospective study, we conducted two sequential experiments. In the factor-quantification experiment, 60 clinically stable neonates in the neonatal intensive care unit (NICU) were monitored simultaneously with radar and a 3-lead ECG bedside monitor as the reference. We assessed HR measurement performance under quiet baseline conditions and four simulated resuscitation-related maneuvers: drying, oronasal suctioning, auscultation, and postural change. Measurement error was quantified using root-mean-square error (RMSE), and agreement was evaluated using Bland-Altman analysis. In the real-workflow experiment, 26 neonates on a radiant warmer in the operating room were enrolled. We analyzed 115 auscultation windows by comparing mean radar-derived HR with auscultated HR at the window level and by stratifying per-second radar-derived HR errors within each window.

**Results:**

Under quiet baseline conditions, radar-derived HR showed agreement with ECG, with most RMSE values below the predefined 10-bpm descriptive benchmark. Measurement performance was relatively preserved during simulated drying, suctioning, and auscultation. In contrast, postural change significantly increased error and caused systematic HR underestimation. In the real-workflow experiment, window-level differences between mean radar-derived HR and auscultated HR within auscultation windows were centered near 0 bpm. In the per-second error analysis within auscultation windows, errors of ≤5 bpm were the most frequent category, whereas errors of >15 bpm were uncommon.

**Conclusion:**

Millimeter-wave MIMO radar showed relatively small HR measurement error for contactless short-window assessment under quiet baseline conditions, particularly when the infant remained stable and oriented toward the sensor. Postural change was the dominant source of increased measurement error and may limit reliability during thoracic reorientation. These findings support the potential role of radar as an adjunct tool for short-window HR assessment during neonatal care and resuscitation-related conditions. Continuous beat-to-beat accuracy was not validated in this study and requires further investigation using continuous ECG-based reference standards.

## 1 Introduction

Neonatal HR assessment is central to perinatal care and decision-making during neonatal resuscitation. Dynamic HR changes guide interventions, reflect physiological transition, and help clinicians evaluate the effectiveness of respiratory and circulatory support (1). Continuous, timely, and reliable HR monitoring can facilitate early recognition of deterioration and improve clinical safety (2, 3). However, conventional methods have well-known limitations in the acute postnatal period.

Three-lead electrocardiography (ECG) provides accurate HR measurements but requires electrode placement, skin preparation, and stable skin contact, which may delay initial readings and increase the risk of skin injury in vulnerable neonates (4–7). Pulse oximetry depends on peripheral perfusion and may provide delayed or unstable readings during the early transitional period. Auscultation is widely used for rapid initial assessment, but it is intermittent, operator-dependent, and unable to provide continuous HR trends (1). In this context, a pre-positioned contactless sensor may be advantageous because HR assessment can be initiated without postnatal attachment of electrodes or probes, thereby reducing interference with early clinical handling and sterile workflow during cesarean delivery or resuscitation-related care.

Millimeter-wave bio-radar represents a promising contactless approach because it can detect cardiac-induced chest wall micromotion without skin contact or electrodes. However, neonatal HR detection remains technically challenging because of the extremely small amplitude of chest wall motion, background interference, and frequent movement during routine care. In particular, the effects of common resuscitation-related maneuvers on radar-derived neonatal HR measurements have not been systematically quantified.

Previous studies have supported the technical feasibility of radar-based monitoring for neonatal vital signs. Kim et al. showed that ultra-wideband (UWB) radar enables reliable detection of neonatal HR and respiratory rate (RR), leveraging its spatial resolution and tissue-penetration capability (8, 9). Nevertheless, the generalizability of their findings was limited by a relatively small cohort and short monitoring periods, which restricted evaluation under more complex clinical conditions. Beltrão et al. used a 24-GHz continuous-wave (CW) radar platform combined with non-negative matrix factorization (NMF) to reduce interference from random neonatal body motion and improve RR estimation in preterm infants with small-amplitude, rapid breathing patterns (10). However, HR signals were not extracted from the same radar dataset, limiting its application for simultaneous HR and RR assessment. Edanami et al. applied nonlinear filtering algorithms to separate respiratory and cardiac signals from radar reflections, achieving simultaneous estimation of HR and RR and refining inter-beat interval analysis in the presence of respiratory artifacts and mild body movement (11). Nevertheless, their neonatal experiments were mainly conducted at short distances within incubators, and common high-impact clinical conditions, such as vigorous movement, posture shifts, and external disturbances during routine care or resuscitation-related procedures, were not systematically assessed.

Our group previously developed a wavelet-based adaptive HR extraction algorithm for neonatal radar monitoring, integrating wavelet transform and adaptive curve fitting for radar-derived HR detection (12). The algorithm fits a smooth HR trajectory from the wavelet time-frequency map and helps distinguish cardiac-related signals from abrupt interference caused by random neonatal body motion. This prior work supported the technical feasibility and preliminary performance of the proposed method. However, it did not specifically examine how radar-based HR measurement performance changes during common neonatal resuscitation-related maneuvers or which types of interference have the greatest impact in clinical workflows.

Collectively, existing studies have provided an important technical basis for radar-based neonatal vital sign assessment, but workflow-oriented clinical evidence remains limited. In particular, there is a lack of empirical data quantifying how routine clinical interventions and resuscitation-related procedures affect radar-derived neonatal HR measurements. This evidence gap limits the translation of contactless radar monitoring from technical feasibility to clinical workflow evaluation.

In the present study, we evaluated how common resuscitation-related maneuvers affect radar-derived HR measurement performance and assessed feasibility within clinical auscultation windows during postnatal assessment and resuscitation-related care. We hypothesized that radar-derived HR would show better agreement with reference measurements under stable conditions but would be more affected by marked postural change.

## 2 Materials and methods

### 2.1 Study design

This was a single-center, prospective experimental study designed to evaluate the measurement performance and workflow feasibility of millimeter-wave MIMO radar for contactless neonatal HR assessment. The study comprised two sequential experiments:

(1) a NICU factor-quantification study to quantify radar-derived HR measurement error and agreement under controlled baseline and simulated resuscitation-related conditions; and
(2) a real-workflow study to evaluate feasibility during clinical auscultation windows on the radiant warmer.

In all experiments, the radar-to-chest distance was set at 60 cm, and the radar sensing surface was oriented approximately perpendicular to the chest wall.

### 2.2 Radar system

The contactless HR assessment system used in this study was a MIMO radar platform adapted from the prototype system developed in our previous work (12). The core radar hardware consisted of a vTrig millimeter-wave sensor evaluation kit (Vayyar Imaging Ltd., Israel), integrated with an onboard MIMO antenna array featuring 20 transmitting antennas and 20 receiving antennas. This system operates as a stepped-frequency continuous-wave (SFCW) MIMO radar, transmitting signals across the 62–69 GHz frequency band. For this study, the operating bandwidth was configured to 1.6 GHz, with a data acquisition frame rate of 30 Hz. The transmitted power was controlled to below −10 dBm, within commonly accepted safety limits for non-ionizing electromagnetic radiation. To ensure reproducible and standardized bedside measurements across experimental conditions, the radar unit was mounted on a mobile adjustable stand using a predefined configuration for each recording session. The radar sensing surface was directed toward the neonatal anterior chest wall, the antenna plane was kept approximately perpendicular to the chest surface, and the radar-to-chest distance was set at 60 cm. This fixed setup was used to standardize signal acquisition across participants and conditions. The predefined configuration was selected based on preliminary setup testing and workflow assessment to balance signal acquisition quality with the need to minimize interference with routine neonatal care and resuscitation-related procedures.

Given the potential application of this system in vulnerable neonatal populations, safety considerations were incorporated into the experimental design and implementation. No radar-associated adverse events, physiological disturbances, or skin irritation were observed in the enrolled neonates during the study period, supporting the short-term tolerability of this contactless measurement setup.

### 2.3 Reference standards and time synchronization

#### 2.3.1 Reference standard in the NICU study (study 1)

In Study 1, a bedside 3-lead ECG monitor (BeneVision N12, Mindray, China) was employed as the continuous clinical reference for simultaneous neonatal HR measurement, allowing comparison with radar-derived HR measurements. Radar signals and ECG waveforms were acquired using an integrated data-recording platform, which was specifically configured to facilitate temporal alignment and comparative analysis between the two modalities. The ECG waveform sampling rate was 500 Hz. To support consistent temporal alignment, both radar output data and ECG monitor signals were recorded with standardized timestamps based on Beijing Standard Time. The two datasets were subsequently matched and aligned on a second-by-second basis using these timestamps, and the ECG-derived HR value corresponding to each Beijing Standard Time timestamp was used as the second-by-second reference for quantitative comparison and measurement-error calculation.

#### 2.3.2 Clinical reference during the real-workflow experiment (study 2)

In Study 2, intermittent HR assessment by auscultation was used as a pragmatic clinical reference during the operating-room radiant warmer workflow. Auscultation was performed by one of three attending pediatricians responsible for neonatal resuscitation at our hospital, according to routine clinical availability, using a hospital-issued Yuwell stethoscope. Radar-derived HR values were not visible to the auscultating clinicians during the assessment. Each discrete auscultation event was defined as an auscultation window, corresponding to the intermittent HR assessment performed during clinical assessment or resuscitation-related care. The start and end timestamps of each auscultation window were identified retrospectively by reviewing video recordings from the fixed routine monitoring camera that documented the clinical workflow.

For each valid auscultation window, the mean radar-derived HR was calculated from second-by-second radar-derived HR estimates and compared with the corresponding auscultated HR. Per-second radar-derived HR errors within each auscultation window, calculated relative to the corresponding auscultated HR, were stratified to describe the short-window error distribution within the clinical workflow.

### 2.4 Participants

#### 2.4.1 NICU cohort (study 1)

Clinically stable neonates admitted to the NICU of Peking University Third Hospital were prospectively enrolled in Study 1. This cohort was selected to enable controlled, standardized evaluation of radar-derived HR assessment in a clinically stable bedside NICU setting. Inclusion criteria were as follows: (i) current admission to the NICU with ongoing routine bedside vital sign monitoring; (ii) baseline clinically stable vital signs with no anticipated acute deterioration, and the ability to maintain hemodynamic and respiratory stability during the data acquisition period; (iii) availability of high-quality, time-synchronized ECG-derived HR reference data for comparative analysis; (iv) no requirement for emergency clinical interventions during the scheduled measurement window that would induce significant fluctuations in neonatal HR.

Exclusion criteria were as follows: (i) severe cardiopulmonary instability or acute clinical decompensation that prohibited completion of data acquisition; (ii) poor-quality reference ECG signals incapable of yielding usable ECG-derived HR reference values; (iii) occurrence of unplanned acute clinical events or therapeutic interventions during data acquisition that would markedly alter vital sign parameters; (iv) persistent radar or ECG monitoring device malfunction, or incomplete data collection that precluded quantitative analysis.

A total of 60 neonates were prospectively enrolled and completed data acquisition in Study 1. The median gestational age at birth was 37.36 weeks (Q1–Q3, 34.39–39.07), and the mean birth weight was 2.62 ± 0.78 kg. All enrolled participants underwent data recording under five predefined, standardized experimental conditions: the no-interference baseline condition, simulated neonatal drying condition, simulated oronasal suctioning condition, simulated auscultation condition, and neonatal posture change condition.

#### 2.4.2 Operating-room cohort (study 2)

Study 2 prospectively enrolled neonates who underwent clinical assessment on a radiant warmer in the operating room immediately following birth. This cohort was designed to reflect the operating-room radiant warmer workflow and evaluate feasibility during clinical auscultation windows. Inclusion criteria were as follows: (i) postnatal clinical assessment or resuscitation-related care conducted on a neonatal radiant warmer within the operating room; (ii) HR auscultation performed by one of the attending pediatricians responsible for neonatal resuscitation during the clinical management; (iii) stable radar operation with complete signal acquisition during the corresponding auscultation periods. Exclusion criteria were as follows: (i) failure to complete required recording due to urgent patient transfer or other workflow-related constraints; and (ii) radar system malfunction during data acquisition.

A total of 26 neonates were enrolled in Study 2. The median gestational age at birth was 38.21 weeks (Q1–Q3, 37.14–38.71), and the mean birth weight was 3.09 ± 0.76 kg. Intermittent auscultated HR values obtained during routine clinical care were documented, resulting in 115 valid auscultation windows for analysis.

### 2.5 Ethics

This study was conducted in accordance with the Declaration of Helsinki and approved by the Medical Research Ethics Committee of Peking University Third Hospital [Approval No. 062-02(2024); date of approval: 1 March 2024]. Written informed consent was obtained from the legal guardians of all participants prior to study enrollment.

### 2.6 Experimental protocols and scenario definitions

#### 2.6.1 Study 1: NICU protocol and predefined conditions

**Figure 1:**
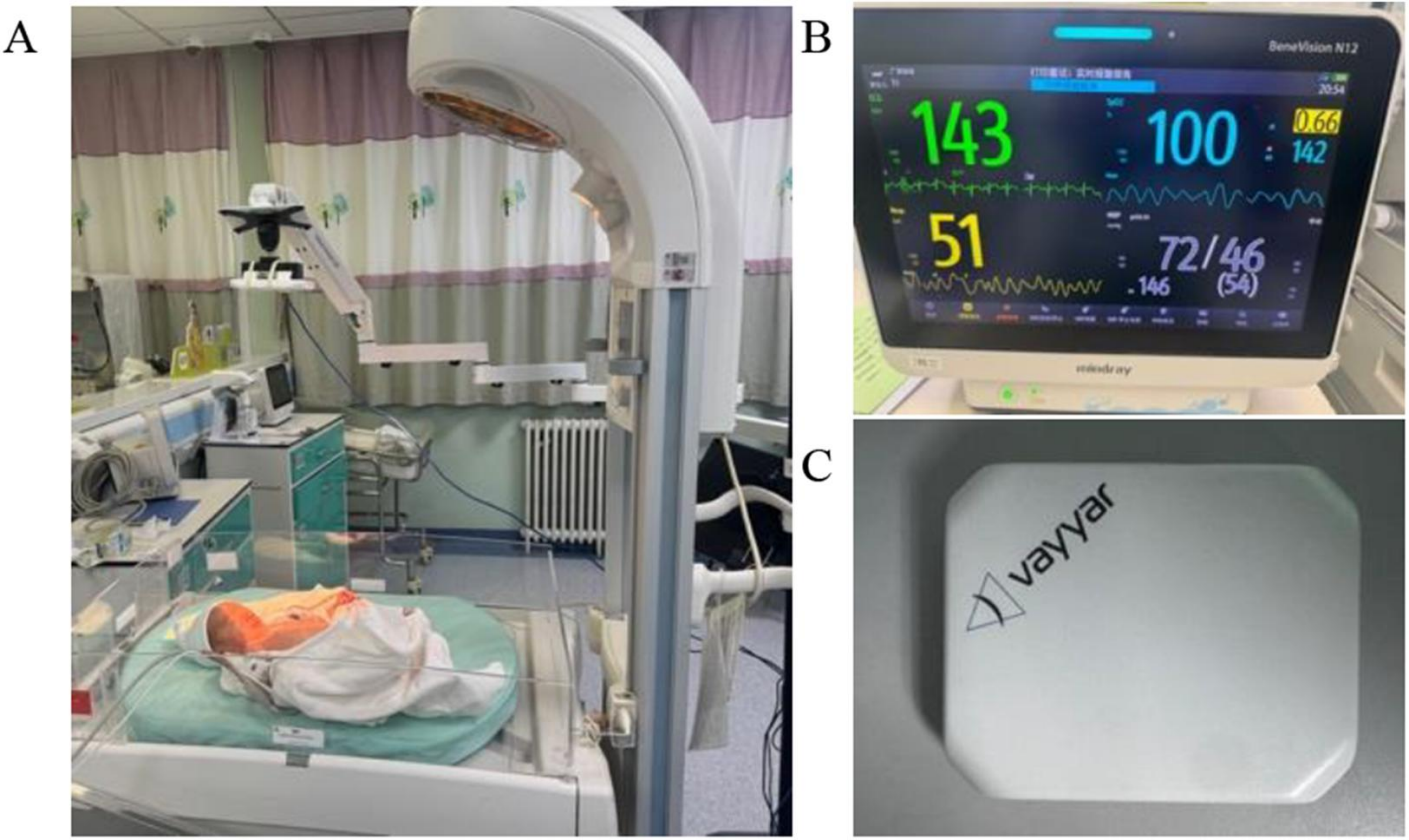
Experimental setup of the neonatal radar monitoring system in the NICU study. **(A)** Overall experimental environment in the neonatal ward, showing the radiant warmer and the radar system mounted on a mobile stand facing the neonatal chest wall; **(B)** Bedside three-lead clinical monitor used as the reference device for heart rate measurement; **(C)** The Vayyar vTrig MIMO millimeter-wave radar sensor used in this study.

Study 1 was designed to quantify the impact of common resuscitation-related maneuvers on radar-derived HR measurement error and agreement under controlled bedside conditions in the NICU. Five predefined study conditions were established, including a no-interference baseline and simulated resuscitation-related maneuvers. The actual data acquisition setup is illustrated in Figure 1.

All recordings in Study 1 were performed using the same standardized radar configuration described above. When clinically feasible, each neonate underwent all five predefined conditions in sequence, with radar and reference data recorded for 60 s per condition.

The five predefined conditions were as follows:

(a) No-interference condition: The neonate remained in a quiet state with no external manipulation. The operator only verified normal device function and refrained from any additional activity near the infant.
(b) Simulated drying condition: Repeated two-hand drying motions were performed above the chest and abdomen to mimic the drying maneuver routinely used in neonatal resuscitation. No direct skin contact was involved. Radar and reference data were recorded for 60 s.
(c) Simulated oronasal suctioning condition: Repeated squeeze-and-release movements of a bulb syringe were performed above the oral and nasal regions to simulate suctioning-related activity during neonatal resuscitation. No direct contact was applied. Radar and reference data were recorded for 60 s.
(d) Simulated auscultation condition: Repeated cycles of stethoscope placement, brief positioning, and removal were performed within 60 s to simulate routine auscultation during neonatal assessment or resuscitation-related care.
(e) Posture-change condition: The neonate's posture was adjusted every 5 s, with each adjustment inducing an approximately 90° change in chest-wall orientation. This condition was designed to represent a predefined large postural perturbation during resuscitation-related handling. Radar and reference data were recorded for 60 s.

#### 2.6.2 Study 2: auscultation-window protocol during postnatal assessment and resuscitation-related care

**Figure 2:**
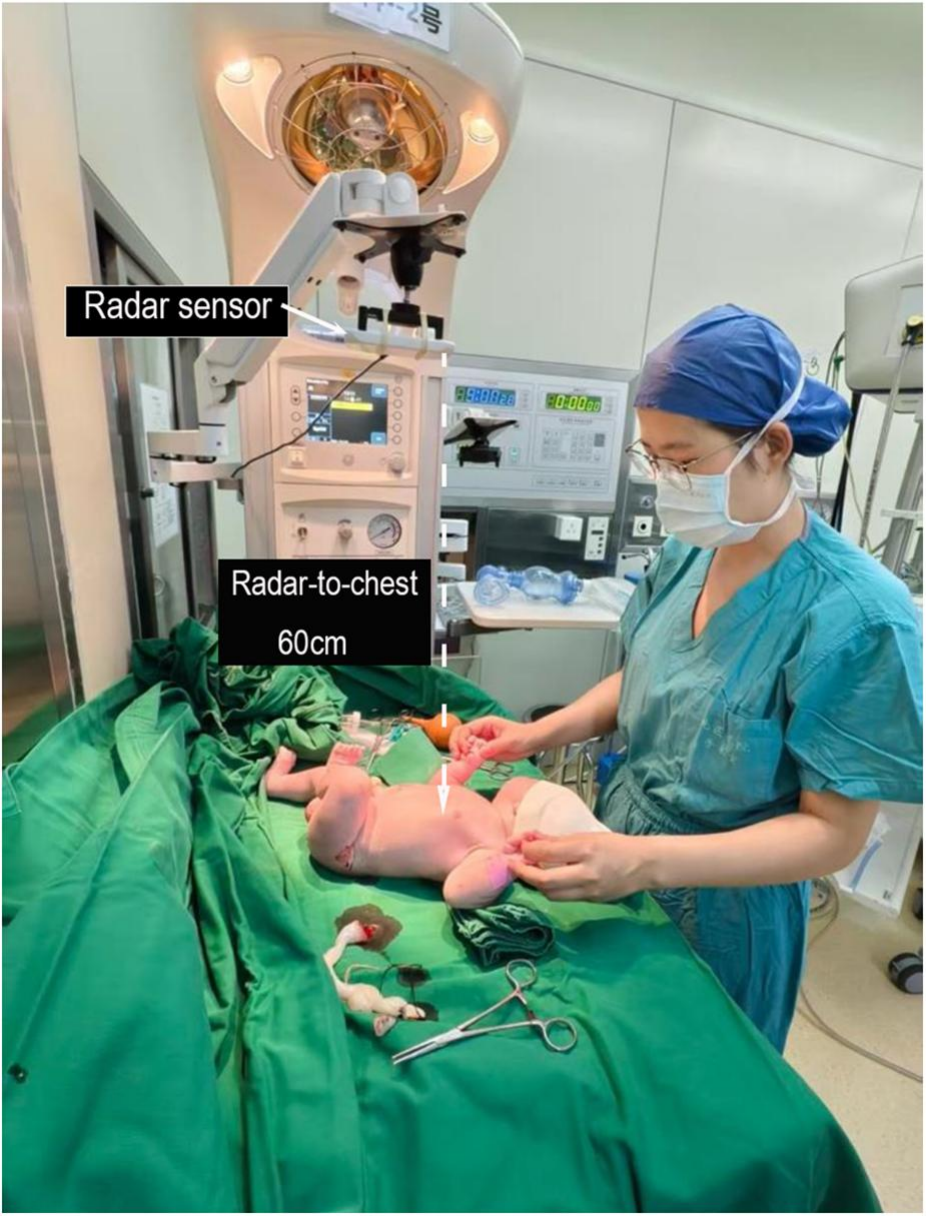
Experimental setup for radar monitoring during neonatal resuscitation in the operating room. The annotated image shows the millimeter-wave MIMO radar sensor, the neonate on the radiant warmer, the approximate sensing direction, and the 60 cm radar-to-chest distance.

In Study 2, radar data were acquired continuously from the moment the neonate was placed on the radiant warmer until being removed from the warmer. The operating-room radiant warmer setup is illustrated in Figure 2. Radar signal acquisition was maintained throughout postnatal assessment and resuscitation-related care without altering routine clinical care.

For subsequent analysis, each clinically performed auscultation episode was defined as an auscultation window, with the start and end timestamps identified via retrospective review of synchronized video recordings. A total of 115 valid auscultation windows were included in the analysis.

Two complementary analytical approaches were applied within these auscultation windows. First, a window-mean comparison was conducted to compare radar-derived HR with the corresponding auscultated HR at the individual auscultation window level. Second, a second-by-second error stratification analysis was performed within each auscultation window to describe the short-window error distribution during the operating-room radiant warmer workflow.

### 2.7 Data and signal processing

Radar-derived HR was obtained using a signal-processing pipeline established in our previous study (12). Detailed derivations have been reported previously (12). To support reproducibility, the key processing steps are summarized as follows: echo signal preprocessing, static clutter suppression, separation of cardiac and respiratory micro-motion components, HR estimation, and data quality control. All signal processing operations were implemented in MATLAB. The radar system has a real-time HR display function; however, this function was disabled during the present study to avoid influencing clinical auscultation and to maintain a consistent offline analysis workflow. Therefore, the results reported in this study were derived from *post-hoc* processing of recorded radar data. Although the algorithm supports simultaneous separation of cardiac and respiratory components, the present study focused on HR assessment, and respiratory-rate outputs were not analyzed. Statistical analyses were performed using SPSS (version 27) and R (version 4.5.1).

### 2.8 Outcome metrics and statistical analysis

#### 2.8.1 Error metrics

In Study 1, second-by-second error was used as the primary analytical unit. Let HR_radar_ represent the heart rate derived from radar and HR_ref_ represent the reference HR, with both values expressed in beats per minute (bpm).

The signed bias was defined as:

$$bias=\frac{1}{n}\overset{n}{\underset{i=1}{\sum }}(HR_{radar,i}-HR_{ref,i})$$

RMSE was defined as:

$$RMSE=\sqrt{\frac{1}{n}\sum_{i=1}^{n}{\left(HR_{radar,i}-HR_{ref,i}\right)}^{2}}$$

#### 2.8.2 Agreement and error evaluation

Under the no-interference condition, agreement between radar-derived HR and ECG-derived reference HR was assessed using Bland-Altman analysis. Subject-level RMSE values were computed and compared across the five predefined experimental conditions to evaluate the impact of predefined resuscitation-related conditions on radar-derived HR measurement error. An RMSE value of 10 bpm was used as a predefined descriptive benchmark rather than as a formal criterion for clinical equivalence. This benchmark was selected because the radar system was intended for use in resuscitation-related settings, where its HR measurement error should ideally be lower than or comparable to the discrepancy observed between conventional clinical HR assessment methods. Previous neonatal studies in birth and resuscitation-related settings have reported systematic differences between auscultation and ECG-based monitoring, with mean differences of approximately 9–14 bpm and individual-level variation extending to approximately ±15 bpm (13, 14). Therefore, 10 bpm was selected as a pragmatic benchmark for describing radar-derived HR measurement error relative to ECG-derived reference HR. For Bland-Altman analysis, second-by-second paired data points were used to derive the mean difference and corresponding 95% limits of agreement.

#### 2.8.3 Repeated-measures analysis of condition effects in study 1

To compare radar-derived HR measurement error across the five predefined conditions, second-by-second measurement errors were analyzed using a repeated-measures linear mixed model. Experimental condition was specified as a fixed effect. Repeated measurements over time_sec were modeled within each participant-condition unit using a diagonal covariance structure. The model was estimated using restricted maximum likelihood, and denominator degrees of freedom were calculated using the Satterthwaite approximation.

When the overall condition effect was statistically significant, pairwise *post-hoc* comparisons were conducted with Bonferroni correction. All statistical tests were two-sided, and *P* < 0.05 was considered statistically significant.

#### 2.8.4 Analysis of auscultation-window data in study 2

In Study 2, each auscultation window was treated as an analysis unit. Descriptive analyses were performed at two hierarchical levels. First, for each auscultation window, the discrepancy between the mean radar-derived HR and the corresponding auscultated HR was calculated to describe window-level difference. Second, the proportion of time falling within each predefined second-by-second error category (≤5, >5–10, >10–15, and >15 bpm) was aggregated across all valid auscultation windows to describe the short-window error distribution during postnatal assessment and resuscitation-related care.

Because clinical auscultation provided an intermittent reference rather than a continuous beat-to-beat reference, analyses in Study 2 were designed to describe radar-derived HR measurements during workflow-based auscultation periods, rather than to validate continuous beat-to-beat accuracy across the full interval between consecutive auscultation assessments.

## 3 Results

### 3.1 Study 1 participants and baseline measurement performance under the no-interference condition

A total of 60 neonates were enrolled in Study 1, and all participants completed data acquisition under all five predefined conditions. The baseline characteristics of the study population are summarized in Table 1.

**Table 1:** Participant characteristics and baseline measurement performance.

Characteristic	Value
Sample size	60
Gestational age at birth (weeks), Median (Q1-Q3)	37.36 (34.39–39.07)
Birth weight (kg), Mean ± SD	2.62 ± 0.78
Postnatal age at examination (days), Median (Q1-Q3)	6 (3.00–14.25)
Body weight at examination (kg), Median (Q1-Q3)	2.56 (2.16–3.21)
Preterm infants	20 (33.33%)
Male	29 (48.33%)

**Figure 3:**
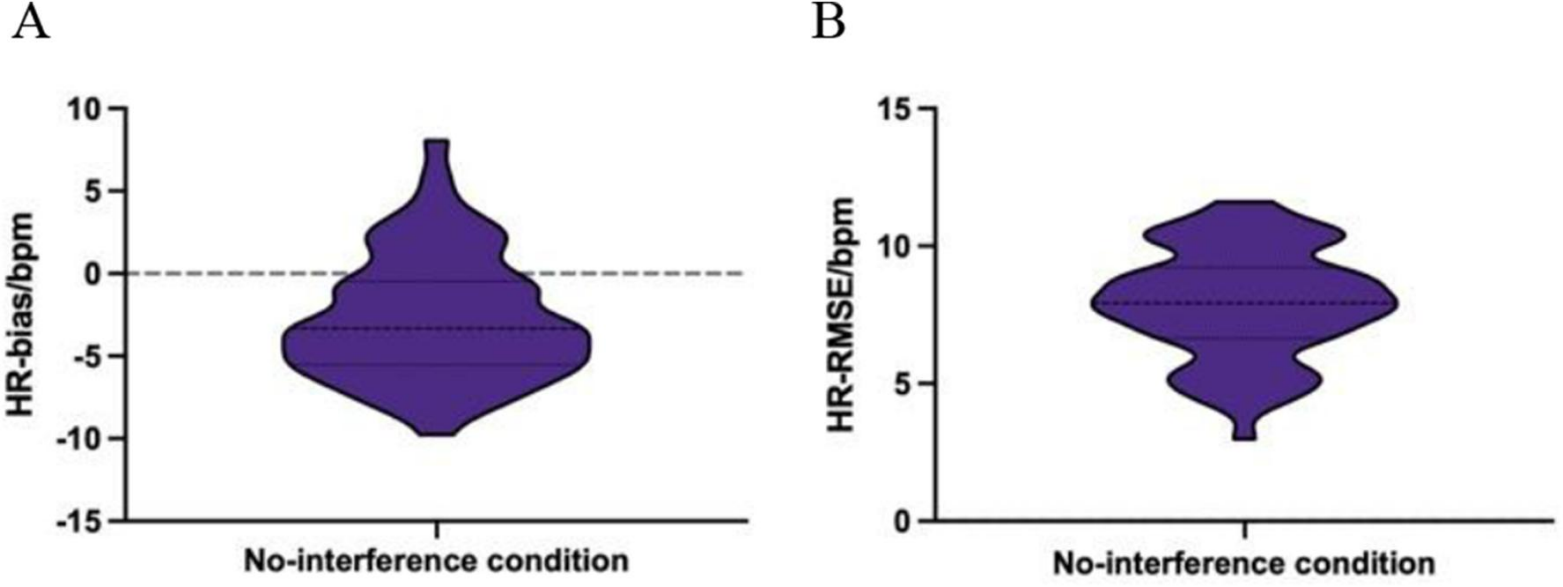
Error distribution of heart rate detection under the no-interference condition. **(A)** Violin plot of bias distribution. **(B)** Distribution of RMSE across participants.

Baseline radar-derived HR was initially evaluated under the no-interference condition. As illustrated in Figure 3(A), the distribution of measurement bias was generally centered near 0 bpm, albeit with a slight tendency toward negative bias. The distribution of RMSE values is presented in Figure 3(B). Most participant-level RMSE values were below the predefined 10 bpm descriptive benchmark, suggesting relatively small participant-level measurement error at the individual level under relatively stable bedside conditions. Nevertheless, a subset of measurements exhibited greater error variability.

**Figure 4:**
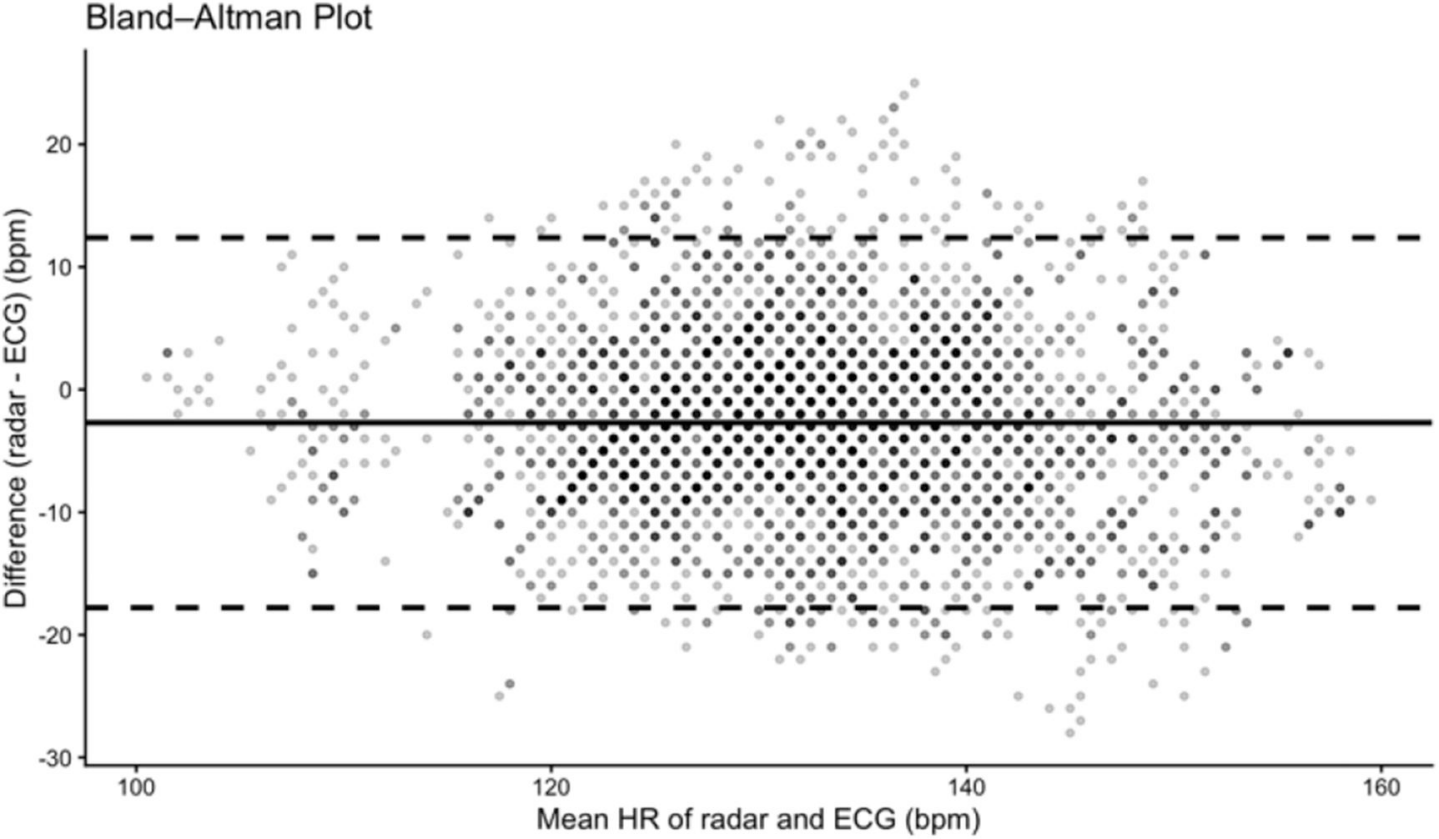
Bland–altman plot showing the agreement between radar-derived HR and ECG monitor HR. The solid line represents the mean bias, and the dashed lines indicate the 95% limits of agreement.

Agreement between radar-derived HR and ECG-derived reference HR under the no-interference condition was further assessed via Bland-Altman analysis (Figure 4). The mean bias (radar-derived HR−ECG-derived reference HR) was −2.702 bpm, with a standard deviation of 7.697 bpm. The 95% limits of agreement ranged from −17.788 to 12.384 bpm. No clear systematic trend was observed across the paired measurements. Among all second-by-second paired observations, 19.36% of samples fell outside the predefined ±10 bpm error band.

### 3.2 Study 1: effects of predefined resuscitation-related conditions on radar-derived HR error

#### 3.2.1 Comparison of error distributions across the five predefined conditions

**Figure 5:**
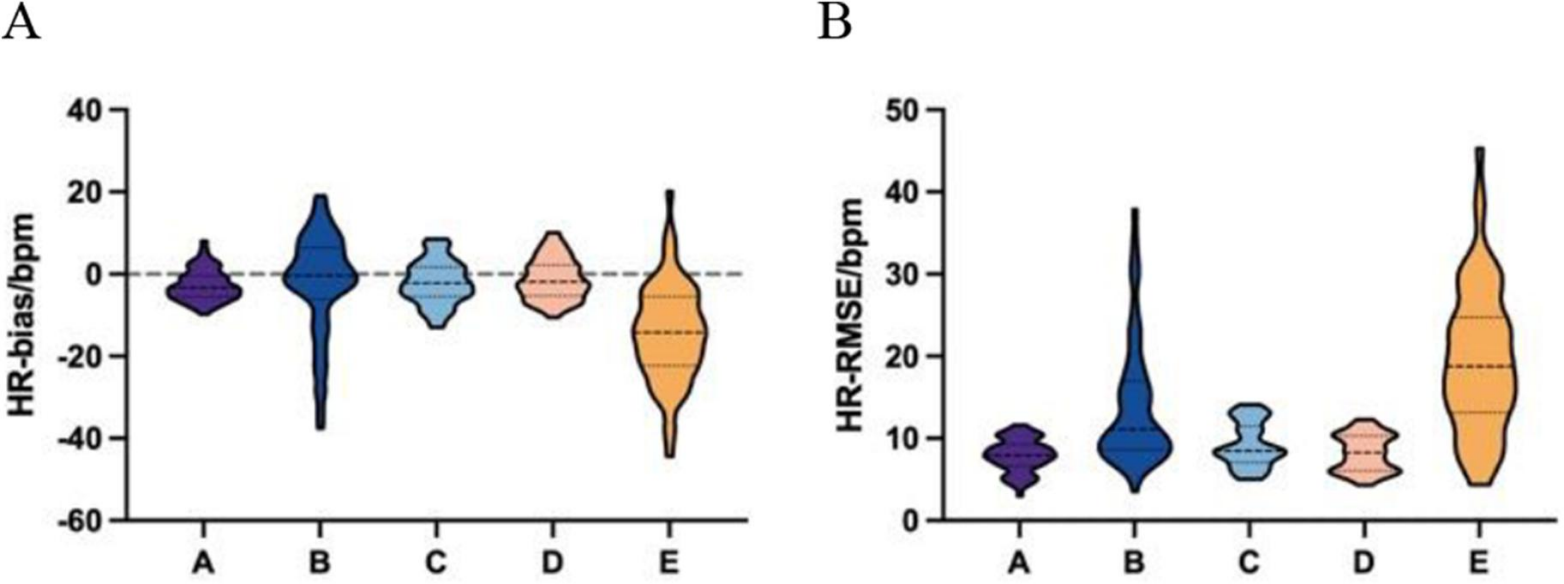
Distribution of heart rate detection errors under five experimental scenarios. **(A)** Bias distributions. **(B)** RMSE distributions. The five scenarios are: **(A)** no-interference condition; **(B)** simulated drying condition; **(C)** simulated oral–nasal suctioning condition; **(D)** simulated auscultation condition; **(E)** posture-change condition.

The distributions of radar-derived HR bias across the five predefined conditions are shown in Figure 5(A). Overall, bias values under the no-interference, simulated drying, and simulated oronasal suctioning conditions were centered near 0 bpm, with limited directional bias. Under the simulated auscultation condition, the bias distribution became slightly narrower and remained close to 0 bpm, indicating modest variability. In contrast, the posture-change condition exhibited a clear shift away from 0 bpm toward negative values, accompanied by substantially increased dispersion. This pattern suggests systematic underestimation of HR during posture change.

The distributions of RMSE across the five predefined conditions are displayed in Figure 5(B). RMSE values under the no-interference, simulated drying, and simulated oronasal suctioning conditions were relatively concentrated with limited overall variability. Under the simulated auscultation condition, RMSE showed a slight increase, yet the overall distribution remained stable. In contrast, the posture-change condition demonstrated a markedly wider RMSE distribution, indicating increased radar-derived HR measurement error under substantial changes in thoracic orientation. Using the predefined 10 bpm descriptive benchmark, participant-level RMSE values were generally below this benchmark under the no-interference, simulated drying, simulated oronasal suctioning, and simulated auscultation conditions, whereas increased measurement error was observed under the posture-change condition.

#### 3.2.2 Linear mixed-effects analysis of condition effects

To further determine whether differences in radar-derived HR error across the five predefined conditions were statistically significant, a linear mixed-effects model was applied. The second-by-second paired difference between radar-derived HR and ECG-derived reference HR served as the primary analysis unit, and the main effect of condition was tested.

Experimental condition exerted a statistically significant effect on radar-derived HR error (F = 802.115, *P* < 0.001). The estimated marginal means were −2.610 bpm for the no-interference condition, −2.077 bpm for the simulated drying condition, −2.160 bpm for the simulated oronasal suctioning condition, −0.972 bpm for the simulated auscultation condition, and −14.444 bpm for the posture-change condition.

Bonferroni-corrected pairwise comparisons revealed that the posture-change condition differed significantly from each of the other four conditions (all *P* < 0.001). The absolute value of the estimated marginal mean under the posture-change condition exceeded the predefined 10 bpm descriptive benchmark, whereas those for the remaining four conditions remained below this benchmark. Significant differences were also detected between the simulated auscultation condition and the no-interference, simulated drying, and simulated oronasal suctioning conditions (all *P* < 0.001). However, the estimated marginal means for these four conditions all remained below the predefined 10 bpm descriptive benchmark. No statistically significant differences were observed among the no-interference, simulated drying, and simulated oronasal suctioning conditions (all *P* > 0.05). Detailed pairwise comparison results are presented in Table 2.

**Table 2:** Bonferroni-corrected pairwise comparisons of heart rate detection differences across experimental scenarios.

Condition 1	Condition 2	*P* -value
no-interference condition	simulated drying condition	0.574
	simulated oral-nasal suctioning condition	1.000
	simulated auscultation condition	＜0.001
	posture-change condition	＜0.001
simulated drying condition	no-interference condition	0.574
	simulated oral-nasal suctioning condition	1.000
	simulated auscultation condition	0.001
	posture-change condition	＜0.001
simulated oral-nasal suctioning condition	no-interference condition	1.000
	simulated drying condition	1.000
	simulated auscultation condition	＜0.001
	posture-change condition	＜0.001
simulated auscultation condition	no-interference condition	＜0.001
	simulated drying condition	0.001
	simulated oral-nasal suctioning condition	＜0.001
	posture-change condition	＜0.001
posture-change condition	no-interference condition	＜0.001
	simulated drying condition	＜0.001
	simulated oral-nasal suctioning condition	＜0.001
	simulated auscultation condition	＜0.001

### 3.3 Study 2: radar-derived HR measurements within clinical auscultation windows during postnatal assessment and resuscitation-related care

#### 3.3.1 Study 2 participants and auscultation-window characteristics

A total of 26 neonates were included in Study 2. Following birth, each neonate was placed on a radiant warmer in the operating room, where postnatal clinical assessment and resuscitation-related care were performed according to clinical need. During this period, HR was intermittently assessed via auscultation by attending pediatricians responsible for neonatal resuscitation. The number of auscultation assessments varied across neonates based on clinical workflow. In total, 115 valid auscultation windows were available for analysis. Baseline characteristics of the Study 2 population are summarized in Table 3.

**Table 3:** Baseline characteristics of neonates included in study 2.

Characteristic	Value
Sample size	26
Birth weight (kg), Mean ± SD	3.09 ± 0.76
Gestational age at birth (weeks), Median (Q1-Q3)	38.21 (37.14–38.71)
Preterm infants	4 (15.38%)
Male	8 (30.77%)

#### 3.3.2 Distribution of mean differences at the auscultation-window level

**Figure 6:**
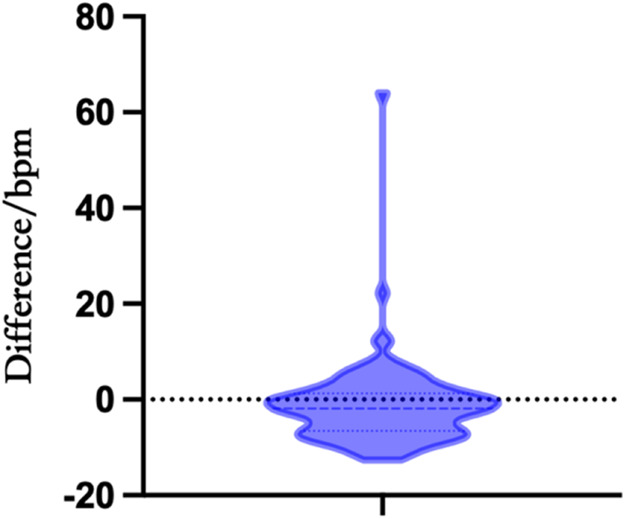
Violin plot showing the distribution of differences between the radar-derived mean heart rate within the auscultation window and the manual auscultation heart rate across 115 auscultation events. The central line represents the median and the dashed lines indicate the interquartile range (IQR).

A total of 115 valid auscultation windows were included in the window-level analysis. For each window, the mean radar-derived HR was calculated from second-by-second radar-derived HR estimates acquired during the corresponding auscultation interval. The difference between this mean radar-derived HR and the corresponding auscultated HR was then used as the analysis unit. The distribution of these window-level differences is shown in Figure 6.

Most differences were concentrated near 0 bpm, with an overall symmetric distribution. This pattern suggested no appreciable systematic bias between radar-derived HR and clinician-auscultated HR during these auscultation windows. The median difference was close to 0 bpm, and the interquartile range was relatively narrow, suggesting relatively small window-level differences. Some windows exhibited larger deviations, indicating larger measurement differences in certain assessment periods, potentially attributable to procedural interference, posture change, or transient motion artifact during the operating-room radiant warmer workflow.

#### 3.3.3 Stratified distribution of second-by-second differences within auscultation windows

**Figure 7:**
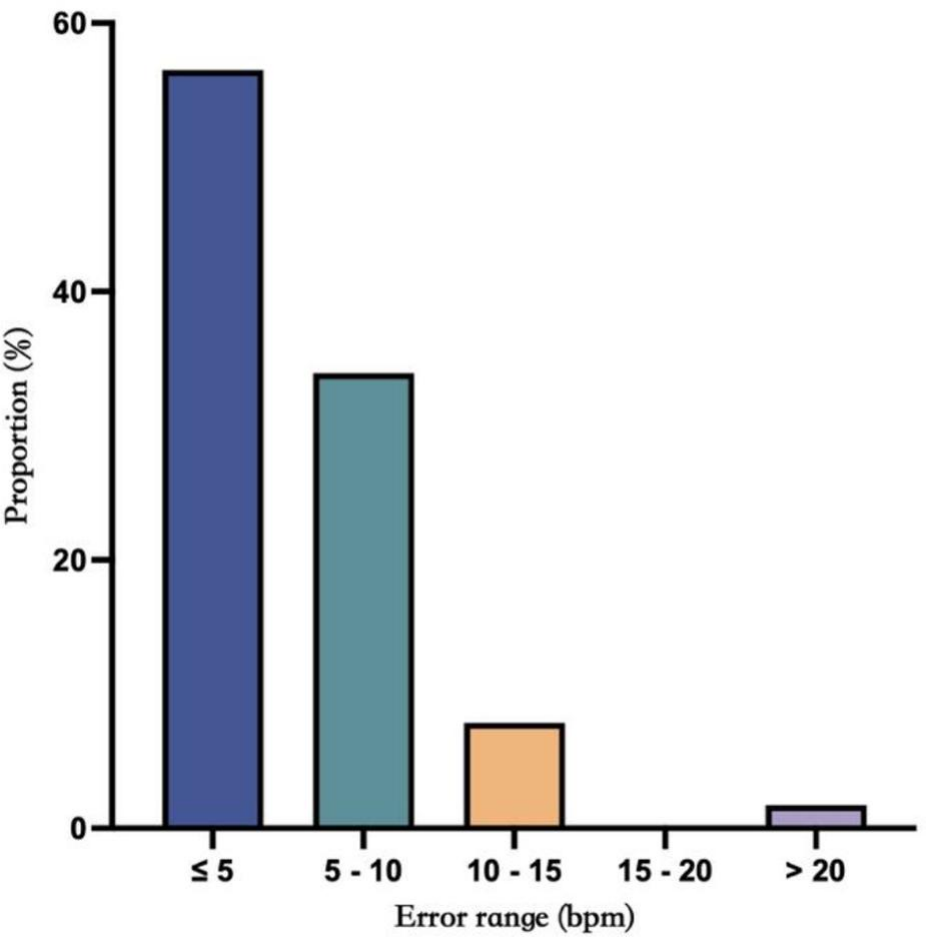
Proportion of second-by-second heart rate differences within the auscultation window stratified by error ranges. The differences were calculated between the radar-derived heart rate and the auscultation value during neonatal resuscitation. Bars represent the proportion of monitoring time within each error range.

To describe the short-window error distribution during the operating-room radiant warmer workflow, second-by-second differences between radar-derived HR and the corresponding auscultated HR were analyzed within each auscultation window. The proportion of time within auscultation windows falling within each predefined error range was subsequently computed, with results displayed in Figure 7.

Differences ≤5 bpm accounted for the largest proportion of time within auscultation windows and represented the dominant component of the distribution. Conversely, the proportion of time with differences >15 bpm was the smallest. Overall, these findings indicate that during most auscultation windows in the operating-room radiant warmer workflow, radar-derived HR showed smaller differences relative to auscultation in most within-window observations. These results support the feasibility of short-window radar-derived HR assessment during workflow-based assessment intervals, while not validating continuous beat-to-beat accuracy across the entire duration between intermittent auscultation assessments.

## 4 Discussion

In the present study, millimeter-wave radar showed relatively small baseline measurement error for contactless HR assessment in neonates under the no-interference condition. Measurement bias was generally centered near 0 bpm with a mild negative trend, and most participant-level RMSE values fell below the predefined 10 bpm descriptive benchmark. Bland-Altman analysis revealed no obvious systematic bias across paired measurements. Collectively, these findings suggest that radar-derived HR showed agreement with ECG-derived reference HR under relatively stable bedside conditions. Notably, however, isolated measurement segments exhibited elevated error levels, suggesting that transient subtle body motion, minor shifts in thoracic orientation, or partial chest occlusion can still increase measurement error even under baseline, undisturbed conditions.

Among the five predefined experimental conditions, the posture-change condition had the most pronounced impact on radar-derived HR measurement error. Relative to the no-interference, simulated drying, simulated oronasal suctioning, and simulated auscultation conditions, posture repositioning induced substantial thoracic reorientation and full-body movement, both of which may affect radar signal acquisition. Such physical changes are likely to disrupt the geometric alignment between the radar sensor and the infant's chest wall, reduce the signal-to-noise ratio of the cardiac micro-motion signal, and amplify motion-related signal contamination. In line with this mechanistic interpretation, the bias distribution under the posture-change condition shifted distinctly toward negative values, while the RMSE distribution widened considerably, indicative HR underestimation and increased measurement error. These observations were supported by linear mixed-effects analysis, which showed that the posture-change condition differed significantly from all the other four conditions (all *P* < 0.001). These results suggest that thoracic alignment and posture stability are more influential determinants of radar-derived HR measurement performance than brief, localized simulated bedside resuscitation-related maneuvers.

Of note, the estimated marginal mean bias under the simulated auscultation condition was closer to 0 bpm than that of several other test conditions. This finding suggests that, under the standardized positioning protocol employed in this study, clinical auscultation itself was not associated with substantial interference with radar-based extraction of chest micro-motion signals linked to cardiac activity. Practically, localized, brief chest-area maneuvers were associated with less radar-derived HR measurement error than marked thoracic repositioning. This is clinically relevant given that auscultation remains a routine component of neonatal resuscitation assessments, and it supports the feasibility of integrating radar-derived HR assessment into clinical workflows involving intermittent auscultation. However, this result should be interpreted as evidence of compatibility within the standardized study setup, rather than conclusive proof that auscultation has no disruptive effect under all real-world clinical scenarios.

Study 2 extended these controlled findings to clinical auscultation windows embedded in postnatal assessment and resuscitation-related care. Within clinical auscultation windows, the distribution of mean differences between radar-derived HR and auscultated HR was centered near 0 bpm and largely symmetric, although some windows showed substantial deviations. Furthermore, stratified analysis of second-by-second differences revealed that measurements with an error of ≤5 bpm accounted for the largest proportion of time within auscultation windows, while larger discrepancies exceeding 15 bpm accounted for the smallest proportion. Taken together, these findings support the feasibility of short-window HR assessment during workflow-aligned auscultation periods. Nevertheless, these results must be interpreted within the limitations of the reference standard: clinical auscultation provides intermittent, clinically meaningful HR data rather than a continuous beat-to-beat reference. Accordingly, Study 2 was designed to describe radar-derived HR measurements specifically during clinical auscultation windows, not to validate continuous beat-to-beat accuracy across the full intervals between consecutive auscultation checks. As such, the present data support the potential role of radar-derived HR assessment as an adjunct to intermittent clinical assessment, rather than as a replacement for auscultation or ECG-based clinical decision-making.

These findings carry several key practical implications for clinical implementation. Radar-derived HR readings showed lower measurement error when the neonate was relatively calm and thoracic orientation remains fixed. Measurements obtained during infant turning or repositioning should be interpreted with caution, as posture change was identified as the primary factor associated with increased measurement error and systematic HR underestimation. These error margins, particularly the negative bias during posture change, support the potential use of radar-derived HR as an adjunct for short-window trend assessment under stable alignment, but they are not sufficient to support its use as the sole basis for life-saving resuscitation decisions. Conversely, brief routine resuscitation-related maneuvers, including drying, oronasal suctioning, and auscultation, were largely compatible with radar-derived HR assessment, although vigorous physical motion or chest occlusion may still degrade signal quality. In future clinical implementation, a pre-positioned contactless radar system may have value during periods when device attachment or repeated manual assessment is difficult, but real-time display performance and workflow integration require further evaluation.

This study has several limitations. First, it was conducted at a single center with a relatively modest sample size, which may limit the generalizability of the findings to broader neonatal populations and diverse clinical settings. In Study 1, clinically stable NICU neonates were evaluated under predefined simulated resuscitation-related conditions. Although this design allowed controlled quantification of specific interference factors, the findings may not fully capture the complexity of active resuscitation scenarios involving unstable neonates, overlapping procedures, and unregulated motion. Second, Study 2 included 26 neonates and 115 auscultation windows and used intermittent auscultation as a pragmatic clinical reference. This approach reflects routine clinical workflow but does not allow continuous beat-to-beat validation throughout the full observation period. Therefore, the Study 2 findings should be interpreted as supporting feasibility within clinical auscultation windows rather than validating continuous radar-derived HR monitoring. Third, the posture-change protocol represented a large predefined postural perturbation, and this study did not determine the angle at which measurement error begins to increase or the time required for signal reacquisition after repositioning. Fourth, the radar system was tested using a standardized fixed measurement geometry, and measurement performance may vary with alternative sensor positions, different room layouts, or clinician movement through the radar field. Finally, although the radar system has a real-time HR display function, this function was disabled in the present study, and the reported results were derived from *post-hoc* processing of recorded data. Respiratory-rate outputs were also not analyzed. Future research incorporating larger multicenter cohorts, more diverse clinical scenarios, continuous ECG-based reference measurements, and real-time workflow evaluation will be essential to further define the clinical role and implementation strategy of radar-based contactless HR assessment in neonates.

## 5 Conclusion

This study evaluated the measurement performance and workflow feasibility of a narrow-beam millimeter-wave MIMO radar system for contactless neonatal HR assessment under controlled simulated resuscitation-related conditions and within clinical auscultation windows during postnatal assessment and resuscitation-related care. Under the no-interference baseline condition, radar-derived HR showed agreement with ECG-derived reference HR. Measurement error remained relatively limited during the simulated drying, simulated oronasal suctioning, and simulated auscultation conditions, while posture repositioning was associated with increased measurement error and HR underestimation. These findings suggest that thoracic orientation and posture stability are important determinants of narrow-beam radar-derived HR measurement performance in neonates.

Within clinical auscultation windows during postnatal assessment and resuscitation-related care, radar-derived HR showed relatively small window-level differences from clinical auscultation, supporting the feasibility of short-window HR assessment during clinical workflow. These results support the potential role of millimeter-wave radar as an adjunctive tool for contactless HR assessment during workflow-based clinical assessment intervals, alongside intermittent clinical auscultation and ECG-based monitoring, rather than as a replacement for established clinical assessment methods. Continuous beat-to-beat accuracy was not validated in this study. Further large-scale, multicenter studies incorporating continuous ECG-based reference measurements and real-time workflow evaluation are required to further validate these findings and define clinical implementation strategies for radar-based HR assessment in neonatal care.

## Data Availability

The datasets presented in this article are not readily available because due to ethical and privacy restrictions involving neonatal clinical data, de-identified data may be made available by the corresponding author upon reasonable request and subject to institutional approval. Requests to access the datasets should be directed to Yanlei Li, liyl002954@aircas.ac.cn.
